# Exercise training improves relaxation response and SOD-1 expression in aortic and mesenteric rings from high caloric diet-fed rats

**DOI:** 10.1186/1472-6793-8-12

**Published:** 2008-05-29

**Authors:** Camila de Moraes, Ana Paula Couto Davel, Luciana Venturini Rossoni, Edson Antunes, Angelina Zanesco

**Affiliations:** 1 Biological Science and Health, Faculty of Physical Education, Cruzeiro do Sul University, São Paulo (SP), Brazil; 2 Department of Physiology and Biophysics, Institute of Biomedical Sciences (ICB-I), University of São Paulo, São Paulo (SP), Brazil; 3 Department of Pharmacology, Faculty of Medical Sciences, University of Campinas, Campinas (SP), Brazil; 4 Department of Physical Education; Institute of Bioscience, University of Sao Paulo State, Rio Claro (SP), Brazil

## Abstract

**Background:**

Obesity has been associated with a variety of disease such as type II diabetes mellitus, arterial hypertension and atherosclerosis. Evidences have shown that exercise training promotes beneficial effects on these disorders, but the underlying mechanisms are not fully understood. The aim of this study was to investigate whether physical preconditioning prevents the deleterious effect of high caloric diet in vascular reactivity of rat aortic and mesenteric rings.

**Methods:**

Male Wistar rats were divided into sedentary (SD); trained (TR); sedentary diet (SDD) and trained diet (TRD) groups. Run training (RT) was performed in sessions of 60 min, 5 days/week for 12 weeks (70–80% VO_2max_). Triglycerides, glucose, insulin and nitrite/nitrate concentrations (NO_x_^-^) were measured. Concentration-response curves to acetylcholine (ACh) and sodium nitroprusside (SNP) were obtained. Expression of Cu/Zn superoxide dismutase (SOD-1) was assessed by Western blotting.

**Results:**

High caloric diet increased triglycerides concentration (SDD: 216 ± 25 mg/dl) and exercise training restored to the baseline value (TRD: 89 ± 9 mg/dl). Physical preconditioning significantly reduced insulin levels in both groups (TR: 0.54 ± 0.1 and TRD: 1.24 ± 0.3 ng/ml) as compared to sedentary animals (SD: 0.87 ± 0.1 and SDD: 2.57 ± 0.3 ng/ml). On the other hand, glucose concentration was slightly increased by high caloric diet, and RT did not modify this parameter (SD: 126 ± 6; TR: 140 ± 8; SDD: 156 ± 8 and TRD 153 ± 9 mg/dl). Neither high caloric diet nor RT modified NO_x_^- ^levels (SD: 27 ± 4; TR: 28 ± 6; SDD: 27 ± 3 and TRD: 30 ± 2 μM). Functional assays showed that high caloric diet impaired the relaxing response to ACh in mesenteric (about 13%), but not in aortic rings. RT improved the relaxing responses to ACh either in aortic (28%, for TR and 16%, to TRD groups) or mesenteric rings (10%, for TR and 17%, to TRD groups) that was accompanied by up-regulation of SOD-1 expression and reduction in triglycerides levels.

**Conclusion:**

The improvement in endothelial function by physical preconditioning in mesenteric and aortic arteries from high caloric fed-rats was directly related to an increase in NO bioavailability to the smooth muscle mostly due to SOD-1 up regulation.

## Background

Obesity is a public health problem and it is a major risk factor for a variety of diseases including arterial hypertension, diabetes mellitus, atherosclerosis and dyslipidemia [1]. Particularly, atherosclerosis is a chronic inflammatory disease associated with endothelium dysfunction and vascular smooth muscle growth resulting in imbalance of vasodilator and vasoconstrictor production by endothelial cells and decrease in vessel lumen [2]. It has been pointed out that oxidative stress associated with alterations in plasma lipid concentration are the main causes of atherosclerosis and both factors play a key role in this cardio-inflammatory disease [3-5].

Evidences have shown that high fat diet provokes alterations in lipid profile and endothelium dysfunction leading to impairment of vascular relaxing responses in rats and pigs [6-9]. A number of studies have reported that the beneficial effect of physical training in lowering LDL-cholesterol and increasing HDL-cholesterol particles [10], as well as improving the endothelium-derived relaxing response and lead to a prevention of atherosclerosis due to increased nitric oxide (NO) production and/or its bioavailability [11-18]. However, no studies exist investigating the effect of run training associated with high caloric diet in the vascular responsiveness in rats.

Therefore, the aim of the present study was to test the hypothesis that prior physical training promotes beneficial effects in the relaxing response of aortic and mesenteric rings from animals fed with high caloric diet and the underlying mechanism mediating this phenomenon. We also evaluated whether run training attenuates the endocrine-metabolic alterations produced by high caloric diet fed-rats.

## Methods

### Animals and experimental protocol

The animal protocols were approved by the Ethics Committee for Experimental Research of the State University of Campinas (UNICAMP). Male Wistar rats (10 weeks-old) were obtained from the Animal Care Facility of UNICAMP. Animals were maintained on a 12 h light/dark cycle, housed in groups of four animals and had free access to water and high caloric diet (56% of carbohydrate, 18% of protein and 26% fat) or standard rat chow (40% carbohydrate, 26.5% protein and 3.8% as fat; Purina Co., Campinas-São Paulo, Brazil). Animals were divided into 4 groups: sedentary control (SD), trained control (TR), sedentary diet (SDD) and trained diet (TRD).

Experimental protocol consisted of 4 weeks of run training program prior to high caloric diet consumption, after which high caloric diet and exercise training were carried out simultaneously for further 8 weeks. Animals were trained in a treadmill with an intensity of 70–80% of maximal oxygen consumption, in sessions of 60 minutes, 5 days a week. Only the animals adapted to the treadmill were used in trained groups. The scheme below illustrates the experimental protocol design, (see Additional File 1).

### Concentration-response curves

Aorta and superior mesenteric arteries were removed carefully and placed in freshly prepared Krebs solution containing (mM): NaCl, 118; NaHCO_3_, 25; glucose, 5.6; KCl, 4.7; KH_2_PO_4_, 1.2; MgSO_4_, 1.17 and CaCl_2_, 2.5. The arteries were cleaned of all adherent tissue and cut into rings of approximately 2 mm. Each ring was suspended between two wire hooks and mounted in 10 ml organ chambers with Krebs solution at 37°C, pH 7.4, and continuously aerated with a mixture of 95% O_2 _and 5% CO_2 _under a resting tension of 10 mN. The tissues isometric tension was recorded by a force-displacement transducer (UgoBasile, Varese, Italy) connected to a PowerLab 400™ data acquisition system (ADInstruments Pty Ltd, Castle Hill, Australia).

After 1 h of stabilization period, intact endothelium aortic and mesenteric rings were pre-contracted with phenylephrine (1 μM). Cumulative concentration-response curves to acetylcholine (ACh, 10 nM-100 μM) and sodium nitroprusside (SNP, 100 pM-100 nM) were obtained in aortic and mesenteric rings with intact endothelium. Concentration-response data were evaluated for a fit to a logistics function in the form:

E = E_max_/((1+(10^c^/10^x^)^n^) + Φ)

where E is the response; E_max _is the maximum response that the agonist can produce; c is the logarithm of the EC_50_, the concentration of agonist that produces half-maximal response; x is the logarithm of the concentration of agonist; the exponential term, n, is a curve fitting parameter that defines the slope of the concentration-response line, and Φ is the response observed in the absence of added agonist. Nonlinear regression analyses to determine the parameters E_max_, log EC_50 _and n were done using GraphPad Prism (GraphPad Software, San Diego-CA, USA) with the constraint that Φ = zero.

### Lipid profile, glucose and insulin concentration

After 12 weeks, animals were sacrificed after an overnight fasting. Blood samples were taken from descendent aorta under anesthesia with pentobarbital sodium (30 mg/kg, i.p.). Plasma and serum were immediately separated by centrifugation (8,000 g). Glucose levels and lipid profile were assessed by using specific commercial kits (colorimetric method, Laborlab, São Paulo, Brazil). Insulin concentration was determined by radioimmunoassay as described previously [19]. Homeostasis Model Assessment (HOMA), an index of insulin resistance was calculated according to a method previously described [20].

### Determination of plasma nitrite/nitrate (NO_x_^-^) levels

In order to evaluate the NO production, the plasma levels of nitrite/nitrate (NO_x_^-^) were measured. Briefly, immediately after arterial blood collecting, the samples were centrifuged (8,000 g) for 10 min, and the resulting plasma supernatant was stored at -80°C. Plasma samples were ultrafiltered through microfilter cups (Microcon Centrifugal Filter Units, 10 kDa; Millipore, Bedford, MA, USA). The NO_x_^- ^concentration of the resulting filtrate solution was determined using a commercially available kit (Cayman Chemical, Ann Arbor, MI, USA) according to the manufacturer's instructions. This assay determines the total NO based on the enzymatic conversion of nitrate to nitrite by nitrate reductase. After the conversion, the spectrophotometric measurement of nitrite is accomplished by using the Griess Reaction. The resulting deep purple azo compound absorbs light at 540–550 nm.

### Western blotting assay for Cu/Zn superoxide dismutase

In order to evaluate the contribution of Cu/Zn superoxide dismutase (SOD-1) in endothelial cells in response to exercise training, aortic and mesenteric expression of SOD-1 was determined by Western blotting assays. Frozen segments of aorta and mesenteric arteries were homogenized and protein concentration was determined using Bradford method [21]. Samples containing 50 μg protein were loaded into gels and eletrophoresed, and proteins were subsequently eletroblotted in polyvinylidene difluoride membranes. Primary antibody was mouse anti Cu/Zn SOD (1:1500, SIGMA, St. Louis, MO, USA). Chemiluminescent signals (ECL plus Amersham, Piscataway, NJ, USA) were captured on X-ray film (Hyperfilm Amersham, Piscataway, NJ, USA), and scanning densitometry was used to quantify the immunoblot signals.

### Drugs and solutions

Acetylcholine, sodium nitroprusside, and phenylephrine were purchased from SIGMA (St. Louis, MO, USA). All other reagents used were of analytical grade.

### Statistical analyses

Data are presented as means ± standard error mean (SEM) of *n *experiments. Comparison of studied parameters and agonist responses was performed by analysis of variance (ANOVA *two-way*) to determine high caloric diet consumption and exercise training interference in results. *Pearson *correlation was used to determine association between triglycerides concentration and endothelium dependent relaxation evoked by acetylcholine. Statistical program SPSS 10.0 was used and the level of statistical significance employed was p < 0.05.

## Results

**Figure 1:**
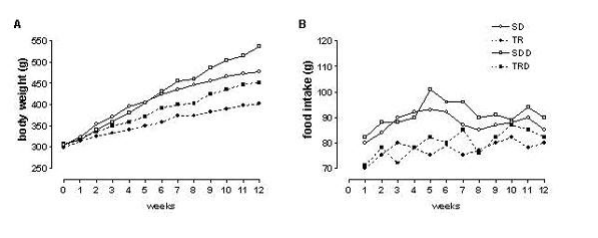
Body weight gain (A) and mean food intake (B) during experimental protocol.

Body weight at initial time of the study was similar in all groups. High caloric diet consumption provoked an increase in body weight gain in SDD group, approximately 13% as compared with SD group. Exercised animals (TR and TRD) showed a smaller weight gain, approximately 16%, as compared with their sedentary groups (SD and SDD). The amount of food intake was also smaller in trained animals (TR and TRD groups) as compared with its matched-sedentary animals (SD and SDD groups). Data are summarized in Table 1 and illustrated in Figure 1.

**Table 1:** Effect of exercise training on body weight, triglycerides, serum glucose, insulin and nitrite/nitrate concentration from animals fed with standard chow or high caloric diet

	**SD**	**TR**	**SDD**	**TRD**
Initial body weight (g)	306 ± 7	297 ± 6	306 ± 8	301 ± 8
Final body weight (g)	478 ± 9	401 ± 8^a^	538 ± 11^c^	452 ± 14^bd^
Triglycerides (mg/dl)	91 ± 11	55 ± 8^a^	216 ± 25^c^	89 ± 9^b ^^d^
Glucose (mg/dl)	126 ± 6	140 ± 8	156 ± 8^c^	153 ± 9
Insulin (ng/ml)	0.87 ± 0.10	0.54 ± 0,10^a^	2.57 ± 0.32^c^	1.24 ± 0.32^b^
HOMA IR	0.61 ± 0.11	0.45 ± 0.09^a^	2.42 ± 0.24^c^	1.08 ± 0.32^b ^^d^
Nitrite/nitrate (μM)	27 ± 4	28 ± 6	27 ± 3	30 ± 2

Data are mean ± S.E.M. for *n *= 8 animals in each group. SD: sedentary, TR: trained, SDD: sedentary diet, TRD: trained diet. ^a ^trained *vs *sedentary; ^b ^trained diet *vs *sedentary diet; ^c ^sedentary diet vs sedentary; ^d ^trained diet *vs *trained.

High caloric diet consumption for 8 weeks provoked a marked increase in triglycerides concentration in SDD group, approximately 138%, which was almost restored to the basal values by exercise training. Furthermore, exercise training per se reduced triglycerides levels, approximately 40% (Table 1). Glucose concentration was significantly greater in SDD group, about 24%, as compared with SD group, and run training did not modify this parameter. Similarly, high caloric diet markedly increased insulin concentration in SDD group, approximately 194% as compared to SD group. Exercise training for 12 weeks attenuated the increase of insulin concentration, approximately 52 % in TRD group as compared with SDD animals. Additionally, exercise training *per se *produced a decrease in insulin concentration (38%) as compared to SD group (Table 1).

In order to verify the insulin resistance, HOMA index was calculated in all groups. High caloric diet produced an increase of HOMA index whereas exercise training alone or associated with high caloric diet consumption produced a significant reduction in this parameter indicating improvement in insulin sensitivity. To evaluate the production of NO metabolites in response to exercise training, the plasma NO_x_^- ^concentration was measured. Our findings showed that NO_x_^- ^levels were not modified in all groups (Table 1).

**Figure 2:**
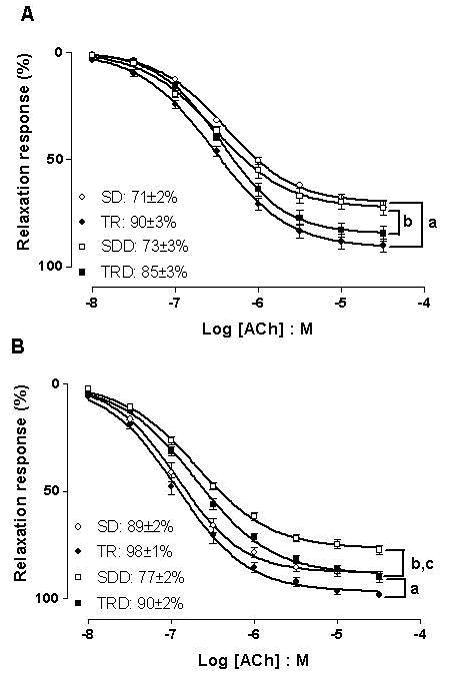
**Concentration response curves to ACh in aortic (A) and mesenteric (B) rings**. Data are means ± SEM of *n *= 5–6 in each group. SD: sedentary, TR: trained, SDD: sedentary diet, TRD: trained diet. ^a ^trained *vs *sedentary; ^b ^trained diet *vs *sedentary diet; ^c ^sedentary diet vs sedentary.

**Figure 3:**
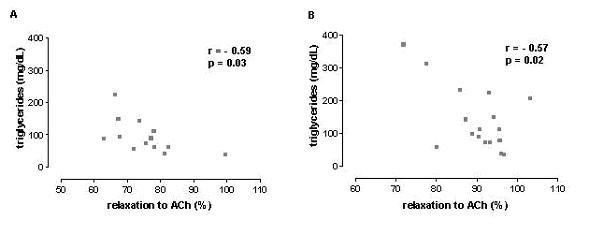
Correlation between triglycerides concentration and maximal relaxation responses to acetylcholine in aortic (A) and mesenteric rings (B).

Endothelium-dependent relaxation responses were evaluated by construction of full concentration-response curves to acetylcholine (ACh) in aortic and mesenteric rings. In aortic rings, run training *per se *produced an increase of the maximal responses to ACh, compared with SD group. Similarly, an increase in the maximal responses to ACh in aortic ring from TRD group was observed. In mesenteric rings, the maximal responses to ACh was reduced in SDD animals while the run training for 12 weeks reversed the impairment of the relaxation responses produced by high caloric diet in TRD group (Figure 2). Similarly, run training alone (TR group) provoked an increase of the maximal responses for ACh (10%). No changes were found in the potency values for this muscarinic agonist in all groups. Data are illustrated in Figure 2. A strong correlation between triglycerides concentration and impairment of relaxing response for ACh was found in both preparations (Figure 3).

**Figure 4:**
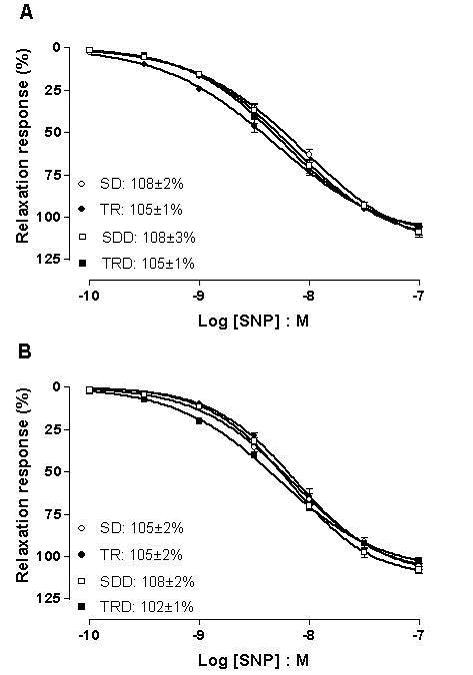
**Concentration response curves to SNP in aortic (A) and mesenteric (B) rings**. Data are means ± SEM of *n *= 5–6 in each group. SD: sedentary, TR: trained, SDD: sedentary diet, TRD: trained diet.

Figure 4 illustrates the concentration-response curves to SNP in aortic and mesenteric rings. This NO donor produced concentration-dependent relaxing responses in both preparations in all groups. Neither maximal responses nor potency values were altered by exercise training (TR group) or high caloric diet consumption (SDD and TRD groups) in both preparations.

**Figure 5:**
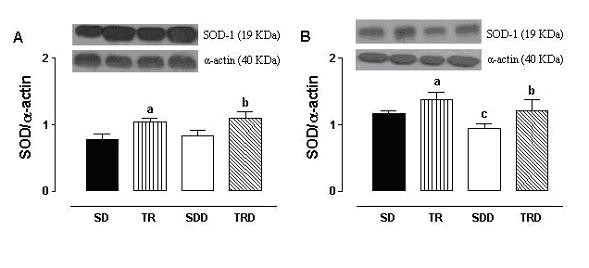
**Effects of exercise training in the SOD-1 expression from isolated rat aorta (panel A) and mesenteric (panel B) arteries**. Representative Western blot (top) and quantitative analysis (bottom) for SOD-1 protein expression. Data are means ± SEM of *n *= 7–8 per group. SD: sedentary, TR: trained, SDD: sedentary diet, TRD: trained diet. ^a ^trained *vs *sedentary; ^b ^trained diet *vs *sedentary diet; ^c ^sedentary diet vs sedentary.

Data from Western blotting analysis in isolated aorta and mesenteric arteries from trained animals fed with standard chow or high caloric diet (TR and TRD groups) showed an increase in the expression of antioxidant enzyme SOD-1, approximately 30%. In mesenteric artery, SOD-1 expression was significantly reduced in SDD animals, but not in aortic rings (Figure 5).

## Discussion

The present study is the first to show that physical preconditioning at moderate intensity restores the impairment of relaxing response in mesenteric rings from high caloric diet-fed rats, which was positively associated with reduction in triglycerides concentration and up-regulation in SOD-1 expression.

Although a variety of experimental models of obesity exist, such as genetic, drugs and diet manipulations, dietary-induced obesity is the most relevant experimental model regarding to human obesity [22]. Our present findings show that run training was effective to reduce the body weight gain and the amount of food intake, indicating that aerobic physical exercise is an important approach in management of both parameters in this particular experimental obesity model.

Impairment of endothelium-dependent relaxation of blood vessels has been consistently demonstrated in a number of studies as a consequence of obesity [6,7,23]. A variety of factors have been proposed to explain the obesity-related endothelial dysfunction including alteration in the adipocyte-derived hormones resistin, adiponectin and leptin [24,25], high plasma triglycerides concentration and increase of oxidative stress [6,7,23,26]. In the present study, high caloric diet significantly produced an impairment of endothelium-dependent relaxation for ACh in mesenteric rings, confirming previous studies.

It is well documented that physical exercise promotes beneficial effects in lipid profile [10,27,28]. Particularly, plasma triglycerides concentration is reduced after exercise training by increasing lipoprotein lipase activity in plasma clearing triglycerides from circulation and replenishing it to the skeletal muscle stores to the process of excitation-contraction coupling (27). Our findings show that physical exercise was efficient to decrease triglycerides concentration after high caloric diet, which was positively correlated with the improvement of maximal relaxation responses to ACh in isolated mesenteric rings. These findings indicate that triglycerides concentration is an important marker for endothelial dysfunction.

High caloric diets are frequently associated with insulin resistance and consequently hyperinsulinemia [29,30]. This condition is related to high level of free fatty acids that increases cellular diacylglycerol leading to activation of different protein kinase C isoforms that phosphorylates serine/threonine sites of insulin receptor (IR) and its substrates. The IR phosphorylation reduces its ability to activate PI3K leading to decrease of the glucose transporters GLUT-4 translocation to the cellular membrane resulting in impairment of glucose uptake [31]. In our study, a marked increase in insulin concentration was found in SDD group that was attenuated after exercise training for 12 weeks, showing clearly the beneficial effects of physical exercise in management of hyperinsulinemia. Similarly, high caloric diet consumption increased blood glucose level, but exercise training failed to restore it to the baseline. The reasons for that could be the magnitude of the alterations in glycemia (approximately of 24%) as compared with insulinemia (approximately 194%) in SDD group. Previous studies have also showed that only a slight increase (or no change) in glucose concentration is seen in dietary-obese rats [30-33].

Either in human or laboratory animals, the cardiovascular benefits of exercise training have been associated with a variety of cellular and molecular alterations including up-regulation of endothelial NO synthase (eNOS), increase in expression and/or activity of antioxidant enzymes, as well as decrease in prooxidant enzyme systems [34,35]. The antioxidant defense systems consist of enzymes such as SOD, catalase and glutathione peroxidase, and non-enzymes including vitamins and flavonoids [36]. The antioxidant enzymes are scavengers of reactive oxygen species (ROS) causing an increase of NO bioavailability to the vascular smooth muscle and enhancement of endothelium-dependent vasodilatation [37-39].

Considering the variety of the receptors and signaling pathways present in the vascular smooth muscle and endothelial cells to trigger the relaxing response, the evaluation of exercise training in the responsiveness of vascular blood vessel is a complex issue. Additionally, multiple interactions exist between the stimulus of an agonist and the vascular response including the affinity of receptor-agonist, metabolism of drugs, existence of antioxidant and prooxidant enzymes in the cell, and the contribution of several protein regulators in the phosphorylation process [35]. At least three isoforms of SOD exist in mammalian tissues, namely Cu/Zn SOD (SOD1), MnSOD (SOD2) and extracellular SOD (EcSOD or SOD3) that are located in cytosol, mitochondria and vascular smooth muscle, respectively [40,41]. Thus, to assess the underlying mechanisms by which exercise training ameliorates the vascular responsiveness in this particular model we have chosen to analyze SOD-1 expression in both arteries and plasma NO_x_^- ^concentration for two reasons. First, SOD-1 represents a major cellular defense against superoxide anion and peroxynitrite formation in endothelial cells. Second, the primary focus of our study was to evaluate the effect of exercise training on endothelium function.

Interestingly, in both aortic and mesenteric rings, an increase in SOD-1 expression in trained groups was found. This up-regulation of SOD was accompanied by an increased in relaxing response for endothelium-dependent agonist. Therefore, our findings clearly show a strong relationship between the improvement in relaxing response seen in trained animals and up-regulation of SOD-1 expression. This is consistent with previous reports in porcine aorta showing that chronic exercise training increases SOD-1 expression associated with improvement in relaxing response [42,43].

In the present study, either high caloric diet or exercise training did not modify the NO production, as estimated by plasma NO_x_^- ^quantification. Previous studies also failed to show a direct correlation between increased NO production and improvement of endothelium-dependent dilation after exercise training [8,42-47], which may be attributed to differences in duration, intensity, and frequency of the training program employed in each study. In fact, to evaluate the effect of exercise training on molecular mechanisms, it is important to consider the total volume of an exercise training program, which is based upon the frequency, intensity, and duration. Accordingly, previous study found that four weeks of exercise training produced an increase of NO production that was positively associated with improvement of relaxing response for endothelium-dependent agonist [48].

In conclusion, in our study, the improvement in endothelial function found in both arteries from trained high caloric-fed rats was directly related to an increase in NO bioavailability to the smooth muscle due to SOD-1 up regulation.

## Authors' contributions

CdM conceived of the project, collected and analyzed the data and wrote the manuscript; APCD and LVR participated in the Western blotting analyses; EA provided laboratories conditions and participated in the project conception; AZ participated in the project conception, the study design, data analysis and writing the manuscript.

## Supplementary Material

Additional file 1 Click here for file
