# Supplementary figures and images for: Exercise training improves relaxation response and SOD-1 expression in aortic and mesenteric rings from high caloric diet-fed rats
Source: BMC Physiol. 2008 May 29;8:12. doi: 10.1186/1472-6793-8-12 (PMC2443377; doi:10.1186/1472-6793-8-12)

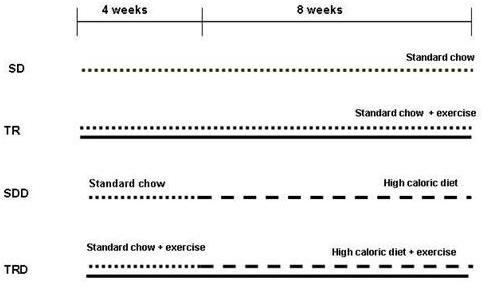

Supplement: Additional file 1 [file 1472-6793-8-12-S1.jpeg]
